# Evolutionary Divergence of Geographic Subspecies within the Scalloped Spiny Lobster *Panulirus homarus* (Linnaeus 1758)

**DOI:** 10.1371/journal.pone.0097247

**Published:** 2014-06-03

**Authors:** Shane D. Lavery, Ahmad Farhadi, Hamid Farahmand, Tin-Yam Chan, Ashkan Azhdehakoshpour, Vibhavari Thakur, Andrew G. Jeffs

**Affiliations:** 1 School of Biological Sciences, University of Auckland, Auckland, New Zealand; 2 Institute of Marine Science, University of Auckland, Auckland, New Zealand; 3 Dept. of Fisheries, University of Tehran, Karaj, Iran; 4 Institute of Marine Biology and Center of Excellence for the Oceans, National Taiwan Ocean University, Keelung 202, Taiwan, R.O.C.; 5 IFRO- Chabahar Branch, Chabahr, Iran; Victoria University Wellington, New Zealand

## Abstract

*Panulirus homarus* is an economically important spiny lobster that is widespread through the Indo-West Pacific Region, but has an uncertain taxonomic status, with three or four geographic subspecies having been described. This study used mitochondrial (16S, COI and control region) and nuclear (18S, ITS-1) DNA sequences to examine specimens of all putative subspecies and forms from throughout their range, in order to determine their genetic validity, and understand the evolutionary history of this species. Despite the range of diversity present in the loci examined, the results were consistent across genes. *P. h. rubellus* from the SW Indian Ocean comprised the most divergent lineage that was reciprocally monophyletic with respect to all other *P. homarus* (approx. 9% divergence in COI), and has likely evolved reproductive barriers. The putative *P. h. “*Brown*”* subspecies from the Marquesas Is in the central Pacific also comprised a somewhat divergent monophyletic lineage (approx. 3% in COI), but may simply be an allopatric population. The widespread *P. h. homarus* was not diverged at all from the described *P. h. megasculpta* from the NW Indian Ocean. The degree of evolutionary divergence of populations at the extremes distribution of the species is somewhat surprising, given the long pelagic larval stage, but suggests that allopatric speciation has been an important driver in the evolution of the genus.

## Introduction

**Figure 1:**
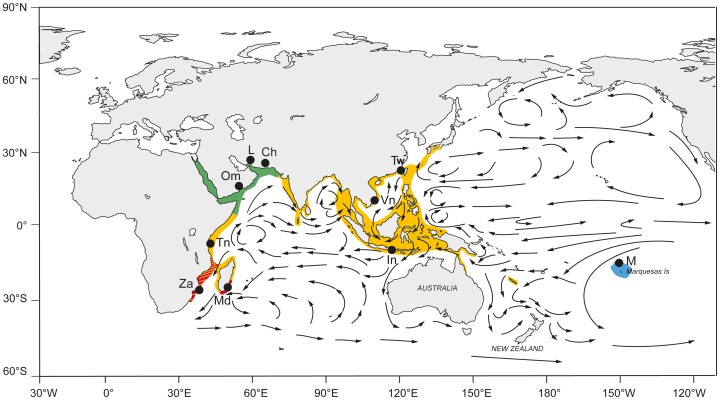
Sampling locations and reported distribution of *Panulirus homarus* and its described subspecies and forms. Yellow: *P. h. homarus*; Red: *P. h. rubellus*; Green: *P. h. megasculpta*; Blue: *P. h.* “Brown”. Sampling location abbreviations are listed in Table 1. The eastern and western limits of *P. h. megasculpta* have not been clearly described. In the SW Indian Ocean, there is uncertainty about the exact distributions of *P. h. rubellus* and *P. h. homarus*, as well as their degree of overlap. Approximate geographic coordinates of sampling locations; Za (−31.880002N, 29.262922E), Md (−25.036189N, 46.983933E), Tn (6.1333SN, 39.3167E), Om (16.947833N, 54.705391E), L (26.854245N, 56.313686E), C (25.348491N 60.512433E), In (−8.942053N, 116.199646E), Vn (16.198191N, 108.247604E), Tw (25.122284N, 121.936569E), M (−8.884732N, −139.998436E).

*Panulirus homarus* (Linnaeus, 1758) is an economically important spiny lobster from the Indo-Pacific region [1]. Despite considerable scientific attention over the years [2], its taxonomic status remains somewhat uncertain, with three or four geographic subspecies or forms having been described [1], [3], [4]. This tropical species is distributed very widely, ranging from the Natal coast of South Africa in the west, to French Polynesia in the east [3] (Figure 1). Recent reports outline a serious decline in the fisheries status of this species throughout its distribution [5], [6] making it more urgent to clarify the ambiguity in its taxonomic status and to assist management processes. The species is also attractive for aquaculture because it is hardy, amenable to culture in sea cages, and grows quickly [7], for which its taxonomic and regional variation is of considerable importance as the basis for selective breeding. Furthermore, an understanding of the origins, evolution and maintenance of any distinct *P. homarus* subspecies will likely provide considerable insight into the important oceanographic and evolutionary forces acting on lobsters and other marine organisms throughout the Indo-West Pacific (IWP) [8]–[10].

**Figure 2:**
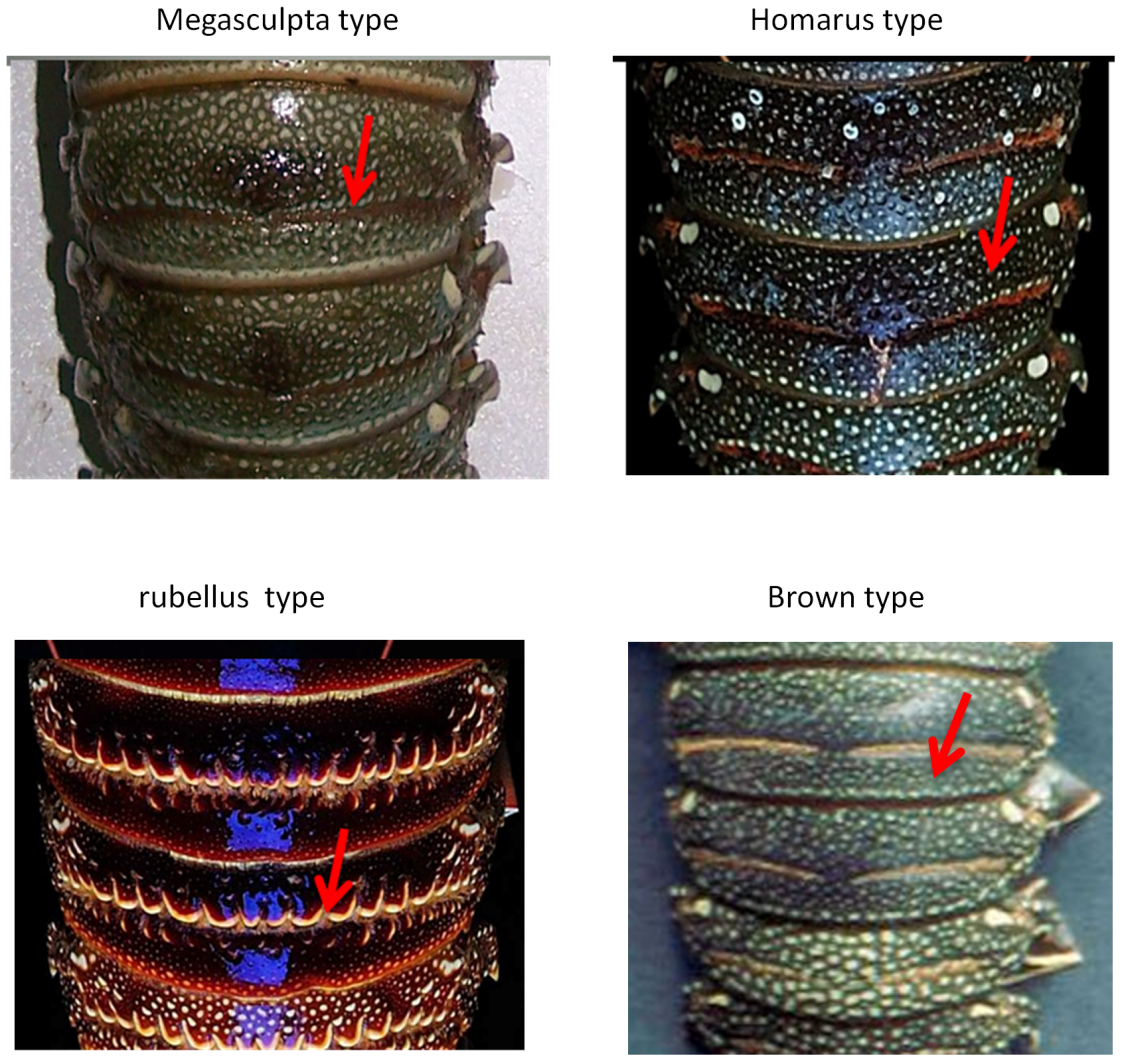
Distinguishing morphological appearances of the subspecies and forms of P. homarus. The colour and sculptus patter are the main morphological identification criteria. The red arrows shows squamae pattern. Rubellus (Madagascar), homarus (Taiwan) and Brown (Marquesas Island) photo; by TYC, megasculpta photo (Chabahar-Iran) by AF.

Based principally on the pattern of sculpturing on the abdomen and colouration, three subspecies have been described for *P.homarus* previously [1], [3], [4]. The nominotypical form *P. homarus homarus* (Linnaeus, 1758) with type locality in Amboina, Indonesia, has small squamae on the transverse abdominal groove, and a mainly green colour, and is believed to occur widely throughout the species' distribution, from South Africa through to the Pacific [3], [4]. *Panulirus h. megasculpta* Pesta 1915 has the type locality in South Yemen, and is distributed in the Arab Sea region. It has large squamae on the abdomen, with yellowish spots on the abdomen, and more or less continuous yellowish lines along the margins of the tergites and pleura. *Panulirus h. rubellus* Berry, 1974 with type locality off the eastern coast of South Africa, with large squamae and brick red colouration, is known only from the South-east African coast and Madagascar [3], [11]. A fourth form has also been proposed from the Marquesas Islands as *P. homarus* “Brown”, which has small squamae and brown colouration (Figure 2). Thus *P. h. rubellus*, *P. h. megasculpta* and *P. h.* “Brown” [4] have geographically discrete distributions, while *P. h. homarus* is described as being widespread (Figure 1). The only genetic investigation so far of variation within this species is that of Ptacek *et al* (2001), who examined two potential subspecies using partial sequences from the 16S rRNA and COI genes. That study described one specimen from the Marquesas Islands and one from Singapore as belonging to *P. h. homarus* and one specimen from Oman as belonging to *P. h. megasculpta*. They found less than 1% sequence difference between these specimens from different subspecies at the 16S rRNA locus but reported 14% sequence divergence in the COI gene between *P. h. megasculpta* and the Marquesas *P. h. homarus* [12]. They concluded that more study was required on the subspecies status of *P. homarus*.

Genetic techniques have successfully been applied to resolving the taxonomic status of many crustacean species [13], [14]–[19]. In particular, mitochondrial 16S rRNA and COI sequences have been used widely in lobster phylogenetic studies [2], and have shown that some other *Panulirus* species are comprised of more than one genetic lineage [16].

To date there has been no comprehensive genetic study focusing on the different subspecies of *P. homarus*. Recent population genetic analysis of samples from the north-west Indian Ocean has revealed some genetic population differentiation within this region [20]. Here, we use DNA sequences from a range of mitochondrial and nuclear genes in all *P. homarus* putative subspecies and forms collected throughout the species' geographic distribution, to test the validity of these previously described subspecies and forms, and to be better understood the species' evolutionary history. We address the following questions: (1) Do the recognized morphological subspecies and forms *P. h. homarus*, *P. h. megasculpta*, *P. h. rubellus* and *P. h.* “Brown” form reciprocally monophyletic lineages indicative of reproductively isolated types? (2) Do any distinct lineages have overlapping distributions, indicating the formation of reproductive barriers between them? (3) Are distinct lineages suggestive of subspecies status, or have they diverged in genetic and morphological characters sufficiently to suggest distinct species status? (4) What do the geographic patterns of genetic divergence suggest about the past evolutionary divergence within *P. homarus*?

## Methods

### Sampling

Specimens of *P. homarus* were collected during 2011–2012 from a range of geographic locations throughout the species' distribution (Figure 1, Table 1). The tissue samples were taken from either identified museum specimens or identified fisheries-caught individuals and were preserved in ethanol until subsequent DNA extraction. The lobster samples from Tanzania and Vietnam were purchased from fishermen, and elsewhere were donated (see Table S1 for details of fisheries). Details of museum specimens sequenced, including institution names, holding locations and specimen catalogue numbers, are provided in Table 1. All other specimens sequenced were sub-sampled from normal fisheries-caught animals, and did not require animal ethics permits, or collecting permits. All fisheries had the appropriate permissions for collections. The species is not endangered or protected. Specimens previously identified by taxonomic experts and/or obtained from Muséum National d'Histoire Naturelle, Paris (MNHN), Natur-Museum Senckenberg, Frankfurt (SMF), University of Florida, Gainesville (FLMNH) and National Taiwan Ocean University, Keelung (NTOU) were also examined by T. Y. Chan and their identity determined in relation to existing morphological descriptions. All museums provided their consent for the use of samples. In the same manner, whole specimens or those with sufficient-quality photographs available were identified by T.Y Chan or Andrew Jeffs based on colour and sculptus pattern. Each putative subspecies and form is represented by multiple individuals from multiple locations, except *P. h.* “Brown”, which is reported from only the Marquesas Islands.

**Table 1:** *P. homarus* and outgroup sampling details and number of individuals (N) sequenced for each locus in this study.

Sample geographic location	Sequence Abbrev.	Collected/Identified by	Sample reference	Morphotype	N				
					COI	16S	CR	ITS-1	18S
South Africa	PhomZa	Fielding, Jeffs	Photos of some	rubellus	5	4	6	6	1
Madagascar	PhomMd	Chan	MNHN^1^	rubellus	3	3	4	2	1
Tanzania	PhomTn	Jeffs	-	homarus	3	1	3	1	-
Oman	PhomOm, PhomChl, Phom06031	Chan, Ptacek	FLMNH^2^ and Ptacek	megasculpta	5	6	6	2	-
Iran-Larak	PhomL	Farhadi, Jeffs	Photos	megasculpta	2	2	3	1	1
Iran Chabahar	PhomC	Farhad, Jeffs	Photos	megasculpta	4	6	6	2	1
Indonesia	PhomIn	Victor Estilo	-	homarus			1		
Vietnam	PhomVn	Ngoc Nguyen Thi	-	homarus	3	3	3	1	-
Taiwan	PhomTw	Chan	NTOU^3^	homarus	2	3	3	1	1
Marquesas Islands	PhomM PhomMLC	Jeffs, Farhadi Chan	Photos MNHN^1^	“Brown”	7	4	7	6	-
Marquesas island	Phom06021	Childress, George	Photos	“Brown”	1	1	1	-	-
Iran – Hangam Isl	PversIr	Farhadi	Photos	*P.versicolor*	-	-	1	-	-
Western Australia	PornChl	Childress	-	*P.ornatus*	-	-	1	-	-

1 Catalogue nos; MNHN 2009-2111, MNHN 2010-4705, MNHN-2010-4706, MNHN 2010-5074. Muséum National d'Histoire Naturelle, Paris, France.

2 Catalogue no; AR15182, Florida Museum of Natural History, University of Florida, Gainesville, FL.

3 Catalogue nos; NTOU M01782, NTOU M01783, NTOU M01784. National Taiwan Ocean University, Keelung, Taiwan.

### DNA extraction and PCR

Whole DNA was extracted from small subsamples of muscle tissue (approx. 50 mg) using a modified phenol-chloroform method [21]. PCR fragments were amplified from two mitochondrial loci 16S rRNA (16S) and cytochrome oxidase subunit I (COI), using the primers 16Sar/16Sbr [22] and LCO1490/HCO2198 [23] respectively. An improved pair of primers for amplifying *P. homarus* COI (LCO-Ph: 5′- TCGGAGCATGAGCTGGGATAGT -3′ and HCO-Ph 5′- ACTTCTGGGTTGTCGAGGACTC-3′) was designed for more consistent amplification and sequencing. Approximately 800 bp of the mtDNA Control Region (CR) was amplified with CRL-F and CRL-R primers [24]. A 1800 bp fragment of the nuclear 18S rRNA (18S) gene was amplified using the primers 18e [25] and 18p [26]. An approximately 800 bp fragment of the nuclear ribosomal complex including the internal transcribed spacer 1 (ITS-1) was amplified using the primers Sp1-5: 5′-CACACCGCCCGTCGCTACTA-3′ and Sp1-3: 5′-ATTTAGCTGCGGTCTTCATC-3′ [27].

All PCR amplifications were undertaken in 25 µl reactions. The reactions contained 2.5 µl of 10× PCR reaction buffer, 2 mM MgCl2, 200 µM of dNTP mix, 0.4 mM primer, 0.125 units of Taq Ti polymerase (Fisher Thermoscientific) and 10–20 ng of total DNA. Reactions were exposed to initial denaturation of 94°C for 4 min, following by 35 cycles of 94°C for 10 s, the respective annealing temperature (59°C for 16S, 62°C for COI, 63°C for ITS-1 and 59°C for CR) for 20 s and 72°C for 30 s, followed by a final extension of 72°C for 5 min. 18S rRNA was amplified with the same conditions except with an annealing temperature of 63C and with 45S of extension in 72°C. All reactions were accompanied by a negative control.

### DNA Sequencing

Free nucleotides and primers were removed from PCR products using a SAP-ExoI protocol [28]. The cleaned products were directly sequenced using the standard protocols of BigDye terminator sequencing chemistry on an ABI PRISM 3100 Genetic Analyser (Perkin-Elmer, Foster City, CA) automated capillary sequencer. Unincorporated dye-labelled nucleotides were removed using the CleanSEQ (Agencourt Bioscience Corporation, Beverly, MA) magnetic bead protocol under recommended conditions. Sequence fragments were generated from approximately 700 bp of the 5′ end of the mitochondrial mtDNA control region (CR), 630 bp of COI, 450 bp of 16S and 1800 bp of 18S, all using the forward primer. Approximately 800 bp of sequence including the ITS-1 were generated using both forward and reverse primers. The heterozygous sequences from individual PhomZa13 were confirmed from multiple PCR products from multiple DNA extractions from that individual. All sequence calls had phred quality scores above 30. Sequences have been submitted to GenBank under accession nos (CR: 906454–906484, COI: KJ802748–KJ802782, 16S: KF923507–KF923532, ITS-1: KJ802725–KJ802747).

### Sequence alignment and phylogenetic analysis

Previously published sequences from additional *Panulirus* species (Table 2) were included in the sequence alignments and phylogenetic analyses to enable comparison of inter- and intra-species divergences. Sequence alignment and editing was undertaken using Geneious v5.6 [29] (MUSCLE alignment [30]) and confirmed by eye. For the CR, sequence divergences beyond the most closely related species were too great to enable confident alignment. For the ITS-1, sequence divergence was too great to successfully align any outgroup species.

**Table 2:** Details of previously published sequences of *Panulirus* species used in phylogenetic analyses.

Species	COI	16S	Location	Ref
*P. h. homarus*	AF339457	AF337963	Singapore, Marq. Isl	[12]
*P. h. megasculpta*	AF339458	AF337961	Oman	[12]
*P. homarus*	JN & JQ^1^	HM & JQ^2^	India	GenBank
*P. argus*	GU476041	JQ412154	North Atlantic	[16]
*P.a. westonii*	GU476045	JQ412156	Brazil	[16]
*P. interruptus*	JN701682	AF337959	California, USA	[12]
*P. gutatus*	AF339456	AF337963	Florida, USA	[12]
*P. penicillatus*-WP	AB610678	AB610710	West Pacific	[56]
*P. penicillatus*-EP	AB610698	AB610717	East Pacific	[56]
*P. echinatus*	AF339454	AF337965	Brazil	[12]
*P. pascuensis*	AF339466	AF337973	Easter Isl	[12]
*P. l. longipes*	AF339464	AF339464	Phillippines	[12]
*P. l. bispinosa*	AF339463	AF339463	Singapore	[12]
*P. cygnus*	AF339453	AF337967	Western Australia	[12]
*P. japonicus*	AF339461	AB610735	Japan	[56]
*P. polyphagus*	AF339469	AF337975	Singapore	[12]
*P. laevicuda*	AF339462	AF337969	Brazil	[12]
*P. regius*	AF339470	AF337976	Congo	[12]
*P. gracilis*	AF339455	AF337964	Mexico	[12]
*P. inflatus*	AF339459	AF337960	Mexico	[12]
*P. marginatus*	AF339465	AF337972	Hawaii	[12]
*P. versicolor*	AF339472	AF337978	Phillippines	[12]
*P. ornatus*	This study	AF337971	Indonesia, Singapore	[12]
*P. stimpsoni*	AF339471	AF337977	Hong Kong	[12]
*Jasus edwardsii*	AF339473	AF337979	New Zealand	[12]

1 -JN418937, JQ229884, JQ229888, JQ229914, JQ229916, JQ229925.

2 - JQ229869, HM015270, HM015271, HM015272.

It is necessary to note that some previously published DNA sequences from *P. homarus* have been found here to be artefacts, and therefore have not been included in the current analyses, and are clearly not reliable for future analyses. The COI sequence of Ptacek *et al.* (2001) from the Marquesas Is (identified there as *P. h. homarus*) (GenBank accession #AF339457) is incorrect, and may be a nuclear pseudogene (or numt), as has been reported from other decapods COI sequences [31]. It is around 22% divergent from all other *P. homarus*, including our samples from the Marquesas, and does not match any other *Panulirus* species. This sequence thus gave a false impression in their work of *P. h. homarus* being very divergent from their *P. h. megasculpta* (Oman) sequence. Similarly, the 18S sequences of *P. homarus* from India reported in [32] appear to be incorrect. These sequences are quite variable among several sampled locations in India, however, they are all quite divergent from our 18S sequences, which show no variation within *P. homarus*.

The best DNA substitution model for each marker was determined by Jmodeltest2 [33], [34]. The models selected were: 16S - HKY+I+G, I = 0.27, G = 0.40; COI - GTR+I+G, I = 0.54, G = 0.62/0.11; CR - GTR+G, G = 0.35; ITS-1 - GTR. Neighbor-joining, maximum likelihood and Bayesian phylogenetic analyses were performed in Geneious, using PHYML [35] and MrBayes [36] with the best substitution models. Between-group average divergences were calculated in Mega 5.2.2 [37].

## Results

As expected, there are considerable differences among genes in the levels of nucleotide diversity within *P. homarus* (Table 3). Nucleotide diversity among all *P. homarus* specimens ranged from 0.0 in 18S to 1.1% in 16S, 5.9% in COI, 8.7% in CR and 2.6% in ITS-1. Although each gene region provides varying detail of the divergence between distinct lineages, all results within *P. homarus* are consistent among loci.

**Table 3:** Average pairwise sequence divergences^1^ (%) between putative subspecies of scalloped spiny lobster (*Panulirus homarus*) (below diagonal) and within-subspecies diversities (on diagonal).

Subspecies	Locus	*P. h. homarus*	*P. h. megasculpta*	*P. h.* “Brown”	*P. h. rubellus*
*P. h. homarus*	16S	0.6			
	COI	1.7			
	CR	6.7			
	ITS-1	0.2			
*P. h. megasculpta*	16S	0.6 (0.2)^2^	0.2		
	COI	1.3 (0.1)	0.6		
	CR	6.1 (0.3)	4.9		
	ITS-1	1.8 (0.6)	2.2		
*P. h.* “Brown”	16S	1.0 (0.6)	0.8 (0.6)	0.1	
	COI	3.1 (1.8)	2.6 (1.9)	0.9	
	CR	11.1 (6.5)	10.5 (6.8)	2.6	
	ITS-1	0.5 (0.5)	2.1 (1.0)	0.0	
*P. h. rubellus*	16S	2.5 (1.9)	2.8 (2.4)	3.0 (2.6)	0.7
	COI	9.0 (7.1)	8.5 (7.2)	9.3 (7.9)	1.9
	CR	31.6 (22.8)	31.4 (23.5)	31.6 (24.8)	9.0
	ITS-1	5.0 (4.8)	3.8 (2.6)	4.4 (4.3)	0.3

1 % sequence divergences calculated using the best model for that locus described in Methods.

2 Net pairwise divergences in brackets.

**Figure 3:**
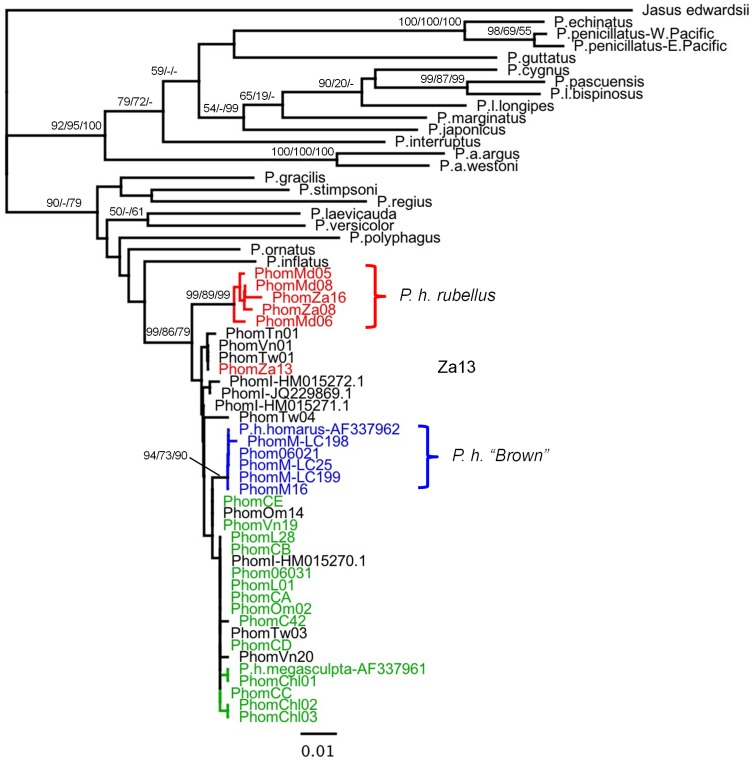
Maximum likelihood phylogenetic tree of 16S sequences from *Panulirus homarus* and other *Panulirus* species. Percent support values (Neighbor-Joining bootstrap/Maximum Likelihood bootstrap/Bayesian probability) shown for nodes with more than 50% support. Sequence location codes are as given in Table 1. Colours are correlated with those in Fig. 1 - Black: *P. h. homarus*; Red: *P. h. rubellus*; Green: *P. h. megasculpta*; Blue: *P. h.* “Brown”.

**Figure 4:**
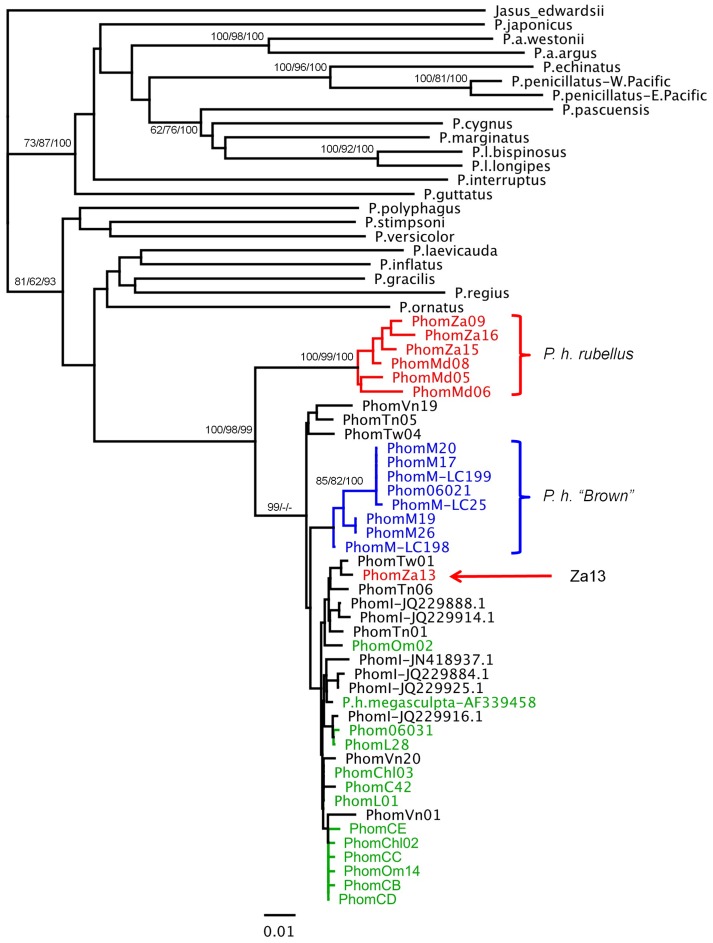
Maximum Likelihood phylogenetic tree of COI sequences from *Panulirus homarus*, and other *Panulirus* species. Percent support values (Neighbor-Joining bootstrap/Maximum Likelihood bootstrap/Bayesian probability) shown for nodes with more than 50% support. Sequence location codes and colours are as in Fig. 2.

**Figure 5:**
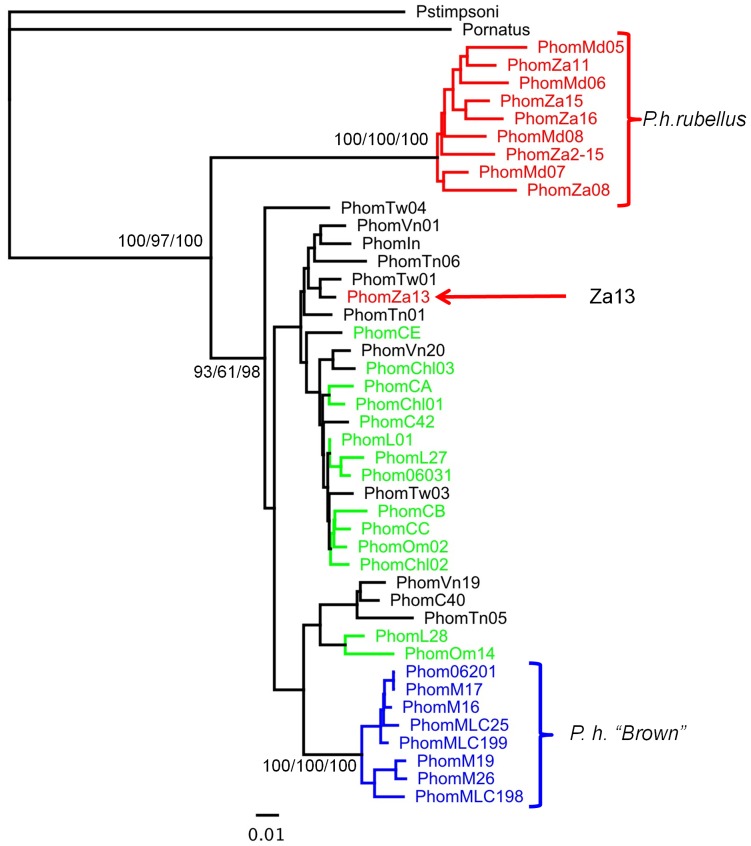
Maximum Likelihood phylogenetic tree of CR sequences from *Panulirus homarus* and outgroup. Percent support values (Neighbor-Joining bootstrap/Maximum Likelihood bootstrap/Bayesian probability) shown for nodes with more than 50% support. Sequence location codes and colours are as in Fig. 2.

**Figure 6:**
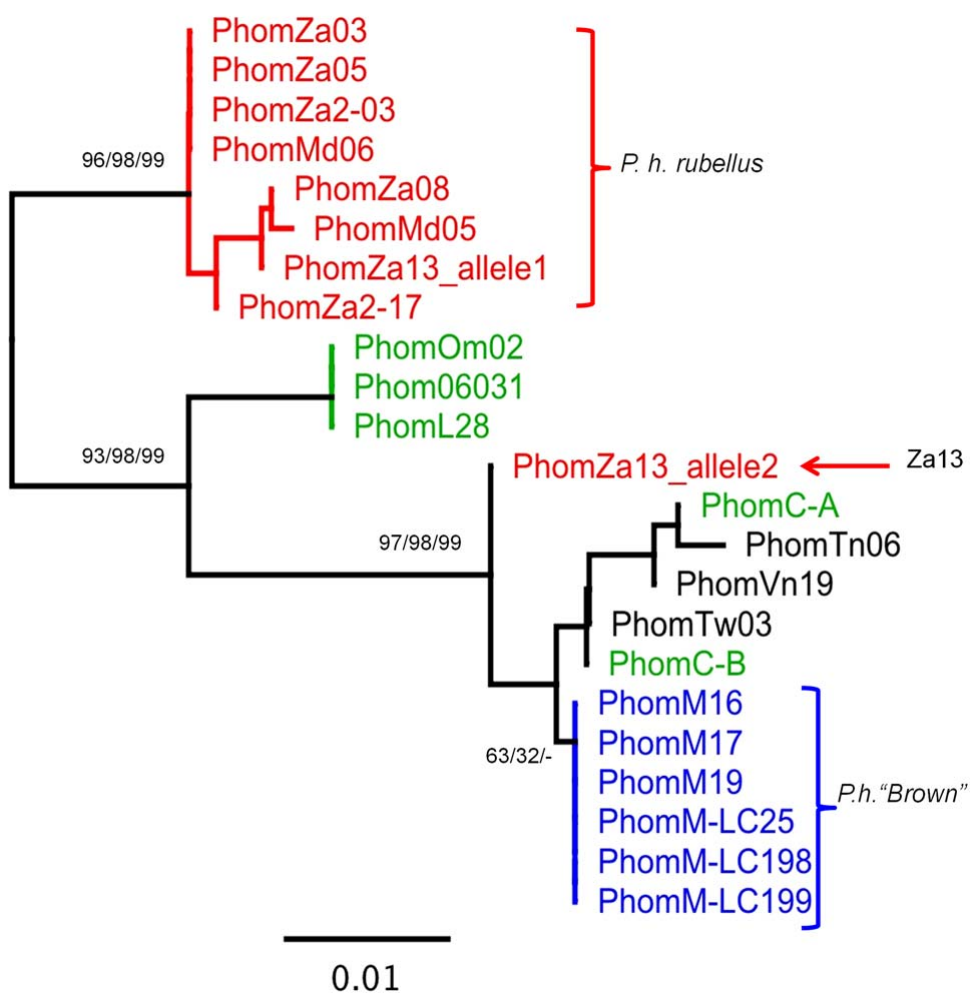
Unrooted Maximum Likelihood phylogenetic tree of ITS-1 partial sequences from *Panuliraus homarus*. Percent support values (Neighbor-Joining bootstrap/Maximum Likelihood bootstrap/Bayesian probability) shown for nodes with more than 50% support.

Phylogenetic trees were constructed for each gene, including sequences available from related species as outgroups, where these could be unambiguously aligned (Figures 3–6). The outgroup sequences allow comparison of within- and between-species divergences. The patterns of relationships among the *P. homarus* individuals belonging to the different putative subspecies and forms are remarkably consistent among loci. The most obvious feature is that there are two major well-supported reciprocally monophyletic lineages observed at each variable locus, representing all specimens of *P. h. rubellus* in one clade and all specimens of all other subspecies and forms in the second clade. This is clearly seen in all of the 16S, COI, CR and ITS-1 phylogenies. The average sequence divergences between the two lineages range between 2.5% in 16S to 31% in the CR (estimated from the best-fit substitution model, accounting for multiple substitutions per site; Table 3). The only individual that does not follow this pattern is PhomZa13. This specimen was collected from South Africa, and identified as *P. h. rubellus*, but has mitochondrial 16S, COI and CR sequences that belong to the other lineage. On examining the ITS-1 sequence derived from this individual, it can be seen that it is heterozygous for two alleles (Figure 6), one that falls into a *P. h. rubellus*–only clade, and the other that falls into the second clade containing all other *P. homarus* specimens. Unfortunately we do not have the whole specimen, or photographs of it, to confirm the morphological appearance of this specimen.

The second most obvious pattern seen in the phylogenies is that all specimens from the Marquesas Islands, and described as *P. h.* “Brown”, also fall into a distinct monophyletic lineage within, and paraphyletic to, the major clade. This lineage is well-supported in all phylogenies except that of ITS-1, where these individuals are all identical to one another, but do not form a distinct well-supported monophyletic lineage. The average sequence divergences between the *P. h.* “Brown” lineage and the *P. h. homarus*/*P. h. megasculpta* lineage range between 0.5% in ITS-1 to 11% in the CR (Table 3).

The remaining sequences of all genes from specimens identified as belonging to the *P. h. homarus* and *P. h. megasculpta* subspecies are found completely mixed within one lineage, and do not show any distinct separation into monophyletic clades. This pattern is repeated in all of the 16S, COI, CR and ITS-1 phylogenies. The net sequence divergences between all *P. h. homarus* specimens and all *P. h. megasculpta* specimens are insignificant for all genes (Table 3).

## Discussion

### Reproductive isolation of subspecies

All the available genetic data shown here from *P. homarus* is consistent in interpretation across the loci. It is apparent that the most distinct lineage within *P. homarus* is that of *P. h. rubellus*. Morphologically, it is readily distinguished from all other subspecies and forms by its red colouring (Figure 2) [3]. The considerably larger squamae on the abdomen also separate *P. h. rubellus* from *P. h. homarus* and *P. h.* “Brown”. Genetically, it clearly belongs to a reciprocally monophyletic lineage that is quite diverged from the remaining *P. homarus* specimens at all variable loci examined (approx. 30% in the CR and 9% in COI). Importantly, this includes divergence at a nuclear locus, ITS-1.

The specimens from the other extreme of the species' range, the Marquesas, also form a distinct monophyletic lineage in 16S, COI and CR phylogenies, but it falls within the major *P. homarus* lineage (i.e., the remaining *P. h. homarus* and *P. h. megasculpta* are paraphyletic with respect to the Marquesas lineage). This Marquesas population has recently been described as another potential subspecies (*P. h.* “Brown”), based on its brownish colouration and remote location [4]. Genetically, it is diverged from the remaining *P. homarus* at most loci examined, but to a much lesser degree than the *P. h. rubellus* lineage (approx. 10% in the CR and 3% in COI). There appears to be insufficient variation at the nuclear ITS-1 locus to unequivocally distinguish the Marquesa specimens as a distinct lineage. Also, there is no clear fixed morphological difference between the Marquesas material and the *P. h. homarus* from Taiwan. Moreover, with additional colouration information now available for both *P. h. homarus* and the Marquesas population, it is clear that there are actually no fixed colouration differences between them. The colouration of the Marquesas specimens is generally olive-green and is simply sometimes lighter (i.e. more greenish) and sometimes darker (i.e. more brownish).

It is also quite clear that the genetic data do not support the separation of the proposed subspecies *P. h. homarus* and *P. h. megasculpta*. Specimens allocated by morphology and geography to these subspecies do not appear to be genetically distinct at all, with the specimens' sequences found mixed throughout the main genetic lineage in each locus examined. There is negligible net sequence divergence between the two putative subspecies at all loci examined. Thus it appears *P. h. megasculpta* should be synonymised with *P. h. homarus*.

These results clearly support the existence of some degree of reproductive isolation among *P. h. rubellus*, *P. h.* “Brown” and the remaining *P. h. homarus*, based on characteristic genetic divergence at mtDNA loci. There is also support from a nuclear locus (ITS-1) for the distinctiveness of *P. h. rubellus*. Existing morphological evidence also supports the distinctiveness of *P. h. rubellus*. The morphological distinctiveness of *P. h. homarus* and *P. h. megasculpta* (based largely on sculpture pattern of abdomen) does not appear to have a clear genetic basis, and there is no evidence of reproductive isolation between these forms.

### Overlapping distributions and reproductive barriers

The four putative subspecies and forms previously described from *P. homarus* have largely non-overlapping geographical distributions. *P. h. rubellus* is reported to occur only in the southwest Indian Ocean (mainly South Africa and Madagascar), *P. h. megasculpta* in the northwest Indian Ocean, and *P. h.* “Brown” from the eastern extreme of the range in the Marquesas. Only *P. h. homarus* has been reported as being widespread through the Indo-West Pacific. Populations of a species can become reproductively isolated over time simply through long-term geographic allopatric separation. Populations reproductively isolated in this way do not necessarily have any biological reproductive barriers between them, and if not, could still successfully reproduce with each other if their gametes meet [38]. If, however, forms that have diverged morphologically and genetically occur in geographic sympatry, then it is likely that there must exist some form of biological reproductive barrier between them at the pre-or post-zygotic stage [39].

Evidence in support of a reproductive barrier with *P. h. rubellus* comes from the maintenance of genetic distinctiveness at both mitochondrial and nuclear genes, in the face of geographic sympatry with the more widespread *P. h. homarus* in at least part of its range. It has previously been reported that along the east African coast (including our sampling site in Tanzania) the common *P. h. homarus* form occurs exclusively as far as the northern Mozambique coast [3]. On the southeast Madagascar coast the *P. h. rubellus* form occurs exclusively [3], [40], whereas along the nearby southeast African coast (including our South African sampling site) both these forms have been reported. Berry recorded that *P. h. rubellus* increases in frequency from approximately 80% of *P. homarus* specimens in southern Mozambique, to over 98% in Natal [3]. The sympatric distribution of two morphological forms that are genetically divergent at both mitochondrial and nuclear genes suggests that these two forms have developed some level of biological reproductive barrier. Their evolutionary divergence appears to have progressed further than merely the stochastic divergence of neutral loci through allopatric separation. Unless there were some reproductive barriers slowing the mixing of their gene pools, the complete monophyletic divergence at neutral genetic loci would rapidly erode with the inevitable gene flow that would arise from sympatric or parapatric distributions. This is particularly so for species such as *P. homarus* with very widely dispersed larvae. The divergent evolution of reproductive structures has previously been suggested to play a role in the development of reproductive barriers between spiny lobster species [41]. Further research could more closely examine differences in reproductive structures between *P. h. rubellus* and *P. h. homarus*.

The extent of the evolutionary divergence between the forms has been clarified to some degree by our genetic studies. One *P. homarus* specimen from South Africa (Za13) has been identified as belonging to the *P. h. homarus* mtDNA lineage (unlike our remaining specimens from that location that belong to the *P. h. rubellus* lineage), whereas this individual is heterozygous in its nuclear ITS-1 alleles, with one allele from the *P. h. rubellus* lineage and one from the *P. h. homarus* lineage. This clearly suggests that this individual is either an F1 hybrid between the two forms, or at least a descendent of one. Thus, although there must likely exist some level of biological reproductive barrier between the forms, for them to maintain their distinctiveness, it clearly must be somewhat permeable. The fact that the hybrid individual possesses *P. h. homarus* mtDNA, indicates that a male *P. h. rubellus* has successfully mated promiscuously with a female *P. h. homarus* when depositing its spermatophores. The extent of hybridisation between the two forms, and whether male *P. h. homarus* can also successfully mate promiscuously with *P. h. rubellus* requires further sampling to clarify. The fact that only one hybrid has been found from our sampling of 44 individuals from South Africa, East Africa and Madagascar suggests an extremely low level of gene flow between *P. h. rubellus* and *P. h. homarus*. The discovery of this hybrid does confirm the suspicions of Berry, who presumed that the occurrence of rare “intermediate” morphotypes in this region indicated the presence of hybrids. He found the frequency of these “hybrids” dropped from 5.1% in southern Mozambique to only 0.1% in Natal [3].

It is also possible that the presence of two divergent alleles (from the *P. h. rubellus* and *P. h. homarus* lineages) in the one individual may be due to either incomplete lineage sorting or past introgression. We consider hybridization to be a more likely cause in this instance, but clearly further investigation is warranted.

### Status of lineages as subspecies or species

The rigorous taxonomic determination of species or subspecies generally requires evidence of distinct morphological and genetic characters, indicating reproductive isolation [42], [43]. We have shown firstly that *P. h. rubellus* is distinctive for morphological colour and genetic characters, and, due to its overlapping distribution with *P. h. homarus*, appears to have some degree of biological reproductive barrier from this form. Furthermore, there is a substantial degree of genetic divergence between *P. h. rubellus* and the other *P. homarus* forms (approx. 30% in the CR and 9% in COI). Thus *P. h. rubellus* warrants at least a distinct subspecies status, and perhaps even a distinct species status. Its genetic divergence from the other *P. homarus* forms is of the same order as that of other described *Panulirus* species. The genetic divergence at COI between species of the genus *Panulirus* varies from approximately 10%, to about 32% [16].

Nevertheless, *P. h. rubellus* still forms a monophyletic clade with the other *P. homarus* forms, indicating its close evolutionary relationship with *P. homarus*. Moreover, the present study shows that *P. h. megasculpta* cannot be separated from *P. h. homarus*. Thus, the larger squamae in *P. h. rubellus* are not useful in differentiating it from *P. h. homarus* and the only reliable distinguishing character is its brick red colouration. However, where both *P. h. rubellus* and *P. h. homarus* occur sympatrically, the two can be readily separated by colouration and size of abdominal squamae. In view of the only distinguishing character of *P. h. rubellus* being colouration (Figure 2), and that natural hybridization occurs between *P. h. rubellus* and *P. h. homarus*, we propose to continue treating *P. h. rubellus* as a subspecies of *P. homarus* until more information on the hybrids is known (e.g. degree of hybridization, fertility of the hybrids, etc.).

*Panulirus h. megasculpta* has previously been reported to occur exclusively in the NW Indian Ocean where no *P. h. rubellus* or *P. h. homarus* morphotypes were found [3]. The Arabian Sea specimens obtained in this study (including four additional specimens from Yemen and Oman deposited in SMF but which could not be successfully sequenced) are all of the *P. h. megasculpta* morphotype. This *Panulirus h. megasculpta* morphotype differs from the *P. h. homarus* morphotype in having bigger squamae and more distinctive yellowish spots on the abdomen (with those on the margins of tergites and pleura often somewhat continuous as a line). However, there is no distinct genetic difference between the Arabian Sea material and *P. h. homarus* in the other Indo-West Pacific localities. Thus the differences in colouration and squamae size do not indicate different genetic lineages or subspecies, and the subspecific status of *P. h. megasculpta* is invalid. It would be very informative to map the exact geographical limitations of the “*Panulirus h. megasculpta*” morphotype as this form appears to be absent in India in the east and Kenya in the west [3], and if both the “*Panulirus h. megasculpta*” and *P. h. homarus* morphotypes can occur sympatrically or not.

The taxonomic status of the *P. h.* “Brown” form is less clear. It is still clearly reproductively isolated from other forms based on its genetic distinctiveness. However, this could be due entirely to its geographic isolation, because there is no fixed morphological and colouration difference between the Marquesas material and other Indo-West Pacific *P. h. homarus*. It is quite possible that no biological reproductive barriers have developed in this form, and it may represent simply a geographically and genetically distinct population of *P. homarus* that has diverged on the eastern periphery of the species' distribution. The fact that *P. h. homarus* mtDNA is paraphyletic with respect to the *P. h.* “Brown” lineage supports the latter being a distinct population rather than a subspecies. The degree of genetic divergence (approx. 10% in the CR and 3% in COI) is similar to that between both distinct subspecies and populations of other *Panulirus* that have been previously described [16]. The taxonomic status of *P. h. “Brown”* can only be finally determined when a greater number of specimens from intermediate localities in the South Pacific islands are included in the analysis.

The gene most used in recent studies for calibrating the age of divergence between crustacean lineages is COI. The most recent and apparently reliable *Panulirus* divergence rate estimate is approximately 1% COI sequence divergence per million years [16], although other estimated rates have been as high as 4% [44]. Using the rate of 1% gives an approximate divergence time for *P. h. rubellus* of about 9 million years ago (MYA), and a divergence time for *P.h.*“Brown” of approximately 3MYA. Using the rate of 4% per MY, gives minimum divergence times of approximately 2.25 MYA and 0.75 MYA, respectively.

### Past evolutionary divergence within *P. homarus*

The *Panulirus* genus is somewhat extraordinary in that most species have relatively long oceanic pelagic larval stages [45], with consequent relatively wide geographic distributions, yet at the same time have undergone extensive evolutionary speciation and radiation [2]. In the Indo-West Pacific alone, there are 12 recognised species of *Panulirus*, many with relatively wide distributions [46]. Given that their long pelagic larval durations would predict relative genetic homogeneity throughout the IWP, the substantial evolutionary radiation in this genus suggests that other mechanisms are involved that restrict successful long-range gene flow through this region. *Panulirus homarus* has one of the wider Indo-West Pacific distributions (although one of the shorter larval durations, at around 6 months) [7], and the evolutionary mechanisms dividing this species into distinct lineages may offer insight into the mechanisms operating throughout this genus to make it such a diverse and successful group.

There are several factors that may potentially limit successful long-range dispersal and/or gene flow in this species. Continental plate movement appears to not have played a role here, as the maximum estimated dates of divergence of the lineages (9MYA) are too recent to be impacted by this, unlike older species divergences in the Palinuridae [47]. Firstly, it is feasible, although not entirely obvious, that the direction of flow of the major currents in the region may have acted to isolate the *P. homarus* populations at the extremes of the species' distribution (Figure 2). Certainly, the predominantly westward flowing currents from the Marquesas would restrict ongoing gene flow in the opposite direction from the distant populations of the western Pacific. The Marquesas are recognised as having a high degree of endemism, particularly in fish and molluscs [9], and its prevailing currents and upwellings have previously been implicated in its genetic isolation [48]. However there is no clear major current directions in the SW Indian Ocean that would isolate this region. It may be more local, less-understood water circulation patterns that play a crucial role here. Of relevance is the apparent genetic divergence of the spiny lobster *Palinurus delagoae* into two distinct lineages (approx. 5% divergent at mtCR) separated by the Mozambique Channel [49]. Discrete SW water movements of the Agulhas and East Madagascar currents may act to minimise larval movement across the Channel [50]. Perhaps more importantly, at both extremes of the *P. homarus* distribution, it is likely that it is the long-term changes in water-circulation patterns that have played the pivotal role. Currents must have been favourable in the past to permit colonisation of these regions by *P. homarus*, and may have subsequently changed for a sufficient period of time to isolate these populations and permit genetic divergence through random drift. Recent currents in the SW Indian Ocean appear to have reduced isolation of the region to some extent, as evidenced by the subspecies' now overlapping distributions, and the occasional hybrid formation. In the Marquesas, perhaps the crucial currents there are eddies and upwelling that permit local retention of larvae after many months [4].

Apart from the influence of currents, other factors are also likely to be important in restricting gene flow in this species. If the larval stages acted as completely passive particles, it is likely that (over their long larval period) they would easily disperse throughout the SW Indian Ocean, breaking down any geographic isolation of populations in this region. It does seem that some level of active movement of the larvae (possibly in response to species-specific orientation cues), in conjunction with the highly active coastward swimming of the postlarval puerulus stage, [51], [52] must restrict dispersal and gene flow to be lower than that expected from currents alone. Several potential behavioural adaptations have been proposed that would help palinurid larvae recruit to local habitat, including diurnal vertical movement [41].

Regardless of the exact mechanisms involved, it is clear that it is only the most geographically extreme populations of *P. homarus* that have diverged to the extent that they may be subspecies. It appears that the most eastern and western populations have diverged relatively recently from the ancestral, central population. This points to allopatric divergence being the dominant evolutionary mechanism in this species. It also suggests that allopatric speciation may be the dominant evolutionary mechanism throughout the genus *Panulirus*. Recent genetic studies have revealed that several described *Panulirus* species are actually comprised of more than one distinct lineage, which have characteristic morphological differences and are now described as either distinct subspecies or species. Most of these distinct lineages are allopatrically distributed. Mitochondrial DNA sequence data was used to propose the divergence of *P. argus argus* from *P. a. westonii* as two subspecies with distinct geographic distributions in the Caribbean and south-west Atlantic respectively [53]. These two clades have recently been confirmed as two monophyletic lineages for both mtDNA genes (16S and COI) and a nuclear gene (ANT) [16]. *P. penicillatus* has been shown recently to consist of two very divergent allopatric lineages from the eastern Pacific and the central and western Pacific [51]. Morphological variants of *P. longipes* have also been confirmed to belong to distinct lineages by mitochondrial COI [54], [55], with two subspecies named *P. l. longipes* and *P. l. bispinosus* (∼3% divergent) that partially overlap in distribution. The closely-related species of the “*japonicus*” group, *P. longipes*, *P. cygnus*, *P. marginatus*, *P. pascuensis*, and *P. japonicus*, all have largely allopatric distributions [55]. This expanding evidence of many closely-related *Panulirus* species and subspecies having non-overlapping distributions highlights the great potential importance of allopatric speciation of peripheral populations in the formation of new genetic lineages within this genus. In the case of *P. h. rubellus*, we appear to have caught this peripheral speciation process in action, as this subspecies has not yet developed a complete reproductive barrier from its sibling, *P. h. homarus*.

### Conclusions

We have shown here using both mtDNA and nDNA sequencing that two of the four putative subspecies in *P. homarus* (*P. h. rubellus* and *P. h.* “Brown”) belong to genetically distinct lineages and are valid taxa, while the remaining two (*P. h. homarus* and *P. h. megasculpta*) are genetically indistinguishable from each other. The partially overlapping distributions of the *P. h. rubellus* & *P. h. homarus* lineages, and the existence of morphological hybrids confirmed genetically by one apparently hybrid specimen in this study, suggest there exists a semi-permeable biological reproductive barrier between these forms. The taxonomic status *P. h. rubellus* is thus valid while that of *P. h. megasculpta* is not. The status of *P. h.* “Brown” remains to be further investigated, particularly with more sampling in the South Pacific, but is clearly reproductively isolated from south-east Asian *P. h. homarus*. Finally, the evolutionary history of this species suggests that it has been dominated by allopatric divergence of populations at the extreme of its distribution. This may point to the principal evolutionary mechanism operating throughout the diverse and successful *Panulirus* genus.

## Supporting Information

Table S1 **Details of sample donor and related fishery organization for permission.**
(DOCX)
