# Supplementary material for: Evolutionary Divergence of Geographic Subspecies within the Scalloped Spiny Lobster Panulirus homarus (Linnaeus 1758)
Source: PLoS One. 2014 Jun 3;9(6):e97247. doi: 10.1371/journal.pone.0097247 (PMC4043530; doi:10.1371/journal.pone.0097247)
Supplement: Table S1 — Details of sample donor and related fishery organization for permission. (DOCX) [file pone.0097247.s001.docx]

| **Sample geographic location** | **Fishery** | **Source status** |  |
| --- | --- | --- | --- |
|  |  |  |  |
| South Africa | Eastern Cape | Donated |  |
| Madagascar | Madagascar | Donated |  |
| Tanzania | Tanzania | Purchased from local fishers |  |
| Oman | Oman | Donated |  |
| Iran-Larak | Iran- Chabahar fisheries Bureau | Donated |  |
| Iran Chabahar | Iran- Bandar Abbas fisheries Bureau | Donated |  |
| Indonesia | Lombok | Donated |  |
| Vietnam | Khanh Hoa | Purchased from local fishers |  |
| Taiwan | Taiwan | Donated |  |
| Marquesas Islands | Marquesas Islands | Donated |  |
| Iran – Hangam Isl | Iran- Bandar Abbas Bureau | Purchased from local fishers |  |
| Western Australia | Northern Western Australia | Donated |  |
|  |  |  |  |

Table S1: Details of sample donor and related fishery organization for permission
